# Zika virus dumbbell-1 structure is critical for sfRNA presence and cytopathic effect during infection

**DOI:** 10.1128/mbio.01108-23

**Published:** 2023-07-07

**Authors:** Monica E. Graham, Camille Merrick, Benjamin M. Akiyama, Matthew J. Szucs, Sarah Leach, Jeffery S. Kieft, J. David Beckham

**Affiliations:** 1 Department of Immunology and Microbiology, University of Colorado School of Medicine, Aurora, Colorado, USA; 2 Department of Medicine, Division of Infectious Diseases, University of Colorado School of Medicine, Aurora, Colorado, USA; 3 Department of Biochemistry and Molecular Genetics, University of Colorado School of Medicine, Aurora, Colorado, USA; Washington University in St. Louis School of Medicine, St. Louis, Missouri, USA

**Keywords:** flavivirus, Zika virus, sfRNA, apoptosis, RNA

## Abstract

All flaviviruses contain conserved RNA structures in the 3′ untranslated region (3′ UTR) that are important for flavivirus RNA replication, translation, and pathogenesis. Flaviviruses like Zika virus (ZIKV) contain multiple conserved RNA structures in the viral 3′ UTR, including the structure known as dumbbell-1 (DB-1). Previous research has shown that the DB-1 structure is important for flavivirus positive-strand genome replication, but the functional role of the flavivirus DB-1 structure and the mechanism by which it contributes to viral pathogenesis are not known. Using the recently solved flavivirus DB RNA structural data, we designed two DB-1 mutant ZIKV infectious clones, termed ZIKV-TL.PK and ZIKV-p.2.5′, which disrupt DB-1 tertiary folding. We found that viral positive-strand genome replication of both ZIKV DB-1 mutant clones is similar to wild-type (WT) ZIKV, but ZIKV DB-1 mutants exhibit significantly decreased cytopathic effect due to reduced caspase-3 activation. We next show that ZIKV DB-1 mutants exhibit decreased levels of sfRNA species compared to ZIKV-WT during infection. However, ZIKV DB-1 mutant 3′ UTRs exhibit unchanged sfRNA biogenesis following XRN1 degradation *in vitro*. We also found that ZIKV DB-1 mutant virus (ZIKV-p.2.5′) exhibited enhanced sensitivity to type I interferon treatment, and both ZIKV-DB-1 mutants exhibit reduced morbidity and mortality due to tissue-specific attenuated viral replication in brain tissue of interferon type I/II receptor knockout mice. We propose that the flavivirus DB-1 RNA structure maintains sfRNA levels during infection despite maintained sfRNA biogenesis, and these results indicate that ZIKV DB-dependent maintenance of sfRNA levels support caspase-3-dependent, cytopathic effect, type I interferon resistance, and viral pathogenesis in mammalian cells and in a ZIKV murine model of disease.

**IMPORTANCE:**

The group of viruses termed flaviviruses cause important disease throughout the world and include dengue virus, Zika virus, Japanese encephalitis virus, and many more. All of these flaviviruses have highly conserved RNA structures in the untranslated regions of the virus genome. One of the shared RNA structures, termed the dumbbell region, is not well studied, but mutations in this region are important for vaccine development. In this study, we made structure-informed targeted mutations in the Zika virus dumbbell region and studied the effect on the virus. We found that Zika virus dumbbell mutants are significantly weakened or attenuated due to a decreased ability to produce non-coding RNA that is needed to support infection, support virus-induced cell death, and support escape from the host immune system. These data show that targeted mutations in the flavivirus dumbbell RNA structure may be an important approach to develop future vaccine candidates.

## INTRODUCTION

The *Flavivirus* genus contains multiple mosquito-borne RNA viruses that impact human health, including West Nile virus (WNV), dengue virus (DENV), yellow fever virus (YFV), and Zika virus (ZIKV) (1). ZIKV, an emerging global pathogen, was first discovered in 1947 in Uganda and has since spread across Africa, southeastern Asia, Micronesia, and South America (2 - 9). It was discovered during the 2015–2016 Brazil outbreak that ZIKV could be transmitted vertically mother to fetus and cause a spectrum of congenital abnormalities referred to as “congenital Zika syndrome” (10). While ZIKV has caused significant outbreaks in more equatorial regions of the world, changing climate and human distribution could expand geographic range and outbreaks in the future (11). With no currently approved antivirals or vaccines, flaviviruses like ZIKV pose a significant risk to human health.

Flaviviruses, including ZIKV, contain in their 5′ and 3′ untranslated regions (UTRs) multiple conserved structural motifs that have evolved to support viral growth and pathogenicity (Fig. 1) (12, 13). Conserved 5′ UTR structures are critical for NS5 binding and activity as well as ribosome subunit positioning around the start codon (12). The 3′ UTR contains several RNA structures with a wide range of functions that include flavivirus non-coding RNA production, *cis*-acting RNA genome interactions that support genome replication, and flavivirus RNA-protein interactions (12, 13). While there is slight variation between individual viruses, the overall RNA structural organization of the 3′ UTR is consistent with one or more three-dimensional (3D) structures known as exonuclease-resistant RNAs (xrRNAs), one or two dumbbell (DB) structures, a short hairpin (sHP), and a 3′ stem loop (3′ SL) (12). At the 5′ end of the 3′ UTR, the xrRNAs are crucial for the biogenesis of sub-genomic flaviviral RNAs (sfRNAs) (12, 14, 15). At the 3′ end of the 3′ UTR, the sHP and 3′ SL play important roles in mediating viral non-structural protein binding to the 3′ end of the genome and 5′ UTR interactions (13). The flavivirus RNA DB structures are located between the xrRNAs and 3′ SL. DENV and WNV contain two complete DB structures, while YFV and ZIKV contain one complete DB and one incomplete DB, referred to herein as a pseudo-dumbbell (12, 16).

**Figure:**
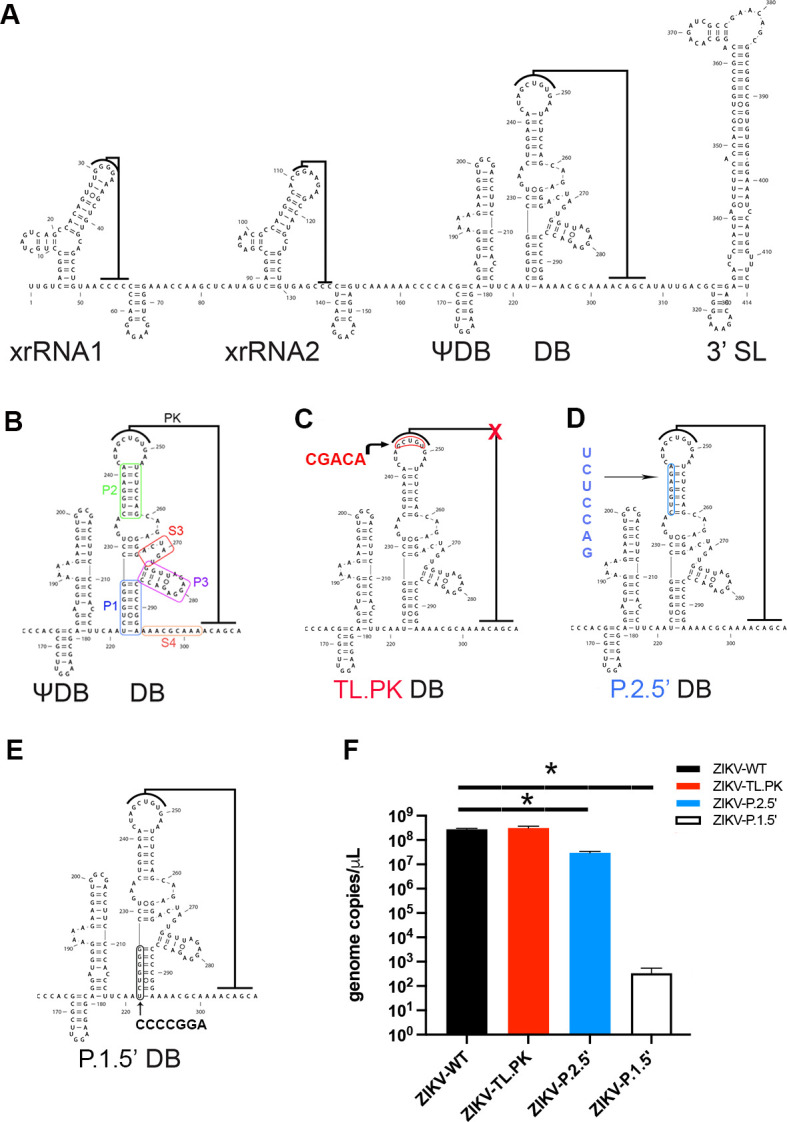
**FIG 1** Zika virus dumbbell mutation design and clone-derived virus rescue. (**A**) Schematic of ZIKV 3′ untranslated region RNA secondary structural organization. (**B**) Individual stem-loop designations in the DB structure. (**C**) Mutational target in the DB structure to create the ZIKV-TL.PK mutant. Red, mutation target. (**D**) Mutational target in the DB structure to create the ZIKV-p.2.5′ mutant. Blue, mutation target. (**E**) Mutational target in the DB structure to create the ZIKV-p.1.5′ mutant. Black, mutation target. (**F**) Genome copies of indicated WT or ZIKV mutant virus in the supernatant following transfection and virus rescue. *P < 0.0004, analysis of variance with Dunnett’s multiple comparisons test. *N* = 3 per group.

Of the flavivirus 3′ UTR RNA structures, the function of the flavivirus DBs is not well understood. One of the current dengue vaccines contains a 30-base pair nucleotide deletion that includes the 5′ end of the DB (17, 18), but the mechanism by which the deletion is attenuating is not defined. In this study, we investigate the contribution of the flavivirus DB-1 structure to flavivirus pathogenesis using ZIKV. We created two mutant ZIKV clones disrupting the integrity of secondary and tertiary DB-1 structural elements. We found that ZIKV-DB1 mutations that disrupt the RNA structure had minimal effect on positive-strand genome replication in mammalian cells, while exhibiting reduced cytopathic effect due to reduced caspase-3 activation. This was associated with a marked reduction in sfRNA levels during ZIKV DB-1 mutant infection in mammalian cells that was independent of sfRNA biogenesis *in vitro*. ZIKV DB-1 mutant decreased sfRNA levels and was also associated with enhanced sensitivity to type I interferon (T1IFN) and a marked increase in survival of ZIKV DB-1 mutant-infected type I/II receptor knockout mice due to reduced replication in brain tissue. Our findings show for the first time that the ZIKV DB-1 RNA structure in the 3′ UTR of flaviviruses functions in part to support sfRNA expression levels after sfRNA biogenesis and contributes to caspase-3 activation, interferon escape, and supports viral pathogenesis in mammalian cells and in a murine model of ZIKV disease.

## RESULTS

### Generation and rescue of ZIKV-TL.PK and ZIKV-p.2.5′ infectious clones

The ZIKV DB-1 structure is predicted to contain a pseudoknot fold, based on the 3D structure of the 3′ UTR dumbbell of the insect-specific Donggang virus and previous work showing conservation of 3′ UTR DB structures in flaviviruses (19 - 22). Specifically, the apical loop on the P2 stem forms Watson-Crick base pairs with downstream 3′ UTR sequence (Fig. 1A and B). In mosquito-borne flaviviruses, this loop pairs with the 3′ cyclization sequence, which can pair with the upstream 5′ cyclization sequence to allow for genome replication (13). Secondary structure analysis has also revealed that the ZIKV DB-1 structure very likely folds the same way as other flaviviral 3′ UTR dumbbells (16, 19). For our first ZIKV mutant clone, termed TL.PK in this study, we mutated the pseudoknot-forming apical loop on the P2 stem (Fig. 1B). We substituted this apical loop sequence with its Watson-Crick complement, eliminating the possibility of pseudoknot base pairing (Fig. 1C). Our second and third ZIKV mutant clones, termed p.1.5′ and p.2.5′, followed the same rationale and design targeting the conserved stems in the DB-1 P1 and P2 stems, respectively (Fig. 1D and E). mFold secondary RNA structure prediction software show that all three ZIKV DB mutations result in significant disruptions to the predicted DB-1 secondary structure (Fig. S1) (23).

From the stock virus created for each ZIKV DB-1 mutant clone, we assessed viral positive-strand genome copy number, infectious titer, and stability of mutations through Vero and C6/36 passaging. Quantifying viral positive-strand genome copy numbers by RT-qPCR revealed that the ZIKV-TL.PK mutant exhibited similar genome copy numbers to ZIKV-WT (Fig. 1F). The ZIKV-p.2.5′ clone exhibited a significant 9.3-fold reduction in positive-strand genome copy numbers compared to ZIKV-WT but still replicated to within a log_10_ of ZIKV-WT and ZIKV-TL.PK. However, the ZIKV-p.1.5′ exhibited mean decrease of 10^6^ positive-strand genome copies compared to ZIKV-WT. Based on these results, we determined that, at least for viral stock generation, the ZIKV-TL.PK and ZIKV-p.2.5′ replicated sufficiently for downstream characterization. However, we determined that the p.1.5′ clone was unable to replicate sufficiently for evaluation in these studies. Following growth of stock ZIKV-WT, ZIKV-TL.PK, and ZIKV-p.2.5′, we sequenced the 3′ UTR and found unchanged mutations in the individual isolates (Fig. S2).

### Viral growth of ZIKV-TL.PK and ZIKV-p.2.5′ infectious clones

To understand potential phenotypic effects of the TL.PK and p.2.5′ mutants, we analyzed the viral kinetics of the ZIKV-TL.PK and ZIKV-p.2.5′ clones. Mammalian A549 cells were infected at an MOI = 0.1, and samples were harvested at 0, 24, 48, and 72 hours post-infection (hpi). We analyzed production of new infectious virus in the supernatant as well as positive-strand ZIKV genome in supernatant and cell-associated samples (Fig. 2). In both supernatant-associated and cell-associated samples, we observed no significant change in ZIKV-TL.PK and ZIKV-p.2.5′ positive-strand genome replication at 24 hpi compared to ZIKV-WT (Fig. 2A and B). Additionally, we found no significant difference in quantity of positive-strand genome replication between our two mutants and ZIKV-WT at 48 and 72 hpi.

**Figure:**
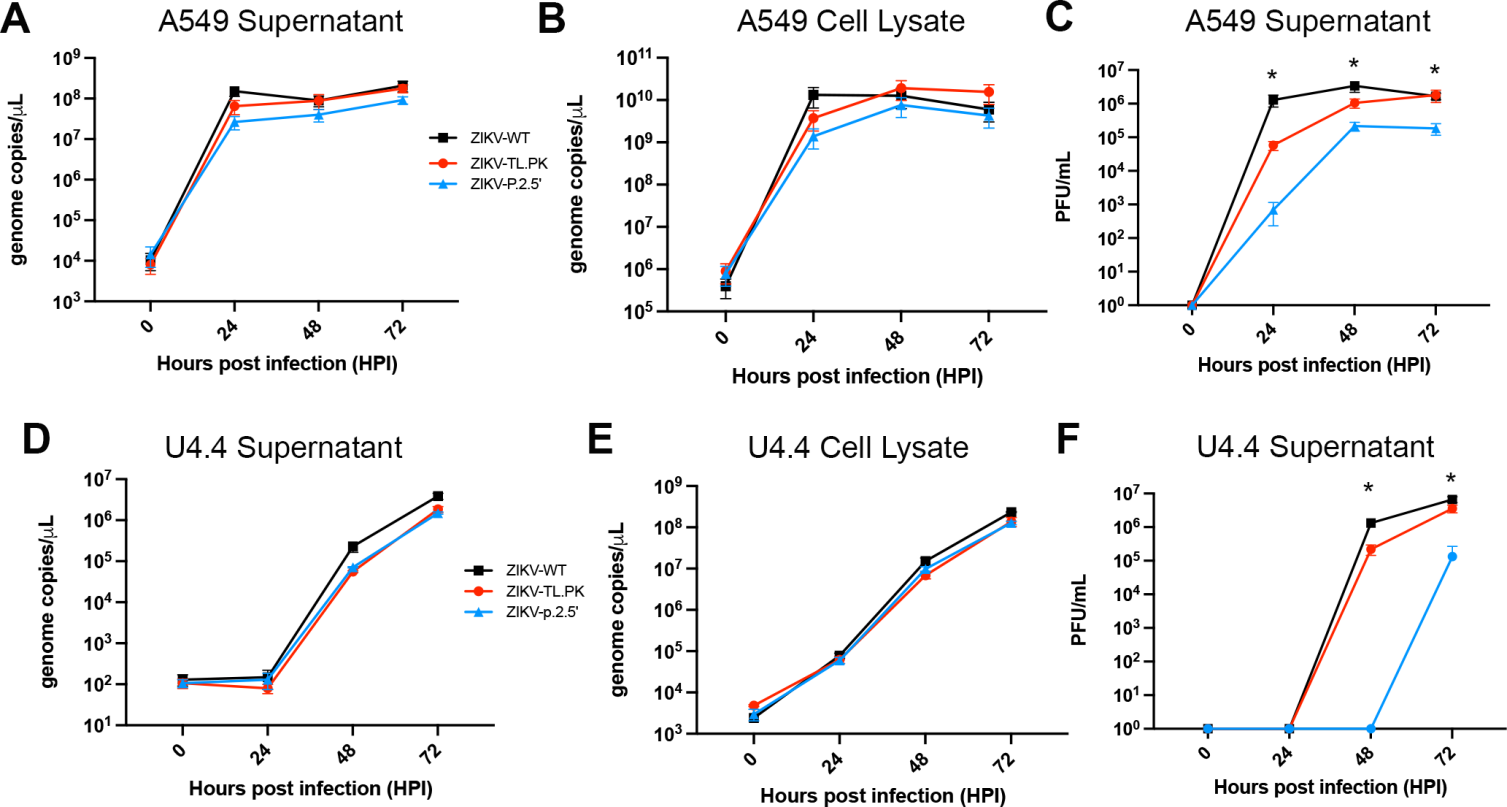
**FIG 2** ZIKV DB mutants exhibit moderately reduced infectious virus titer in cell culture but unchanged viral genome replication. A549 cells were inoculated with ZIKV-WT, ZIKV-TL.PK, and ZIKV-p.2.5′ (MOI = 0.1) followed by harvest of (A) supernatant and (B) A549 cells at the indicated time points post-infection. (**C**) A549 cells were inoculated with ZIKV-WT, ZIKV-TL.PK, and ZIKV-p.2.5′ (MOI = 0.1) followed by harvest of supernatant for plaque assay analysis. *P < 0.0001, Two-way analysis of variance (ANOVA). *N* = 9 total per group/time point, completed as triplicates in three experimental replicates. U4.4 cells were inoculated with ZIKV-WT, ZIKV-TL.PK, and ZIKV-p.2.5′ (MOI = 0.1) followed by harvest of (D) supernatant and (E) U4.4 cells at the indicated time points post-infection. (**F**) U4.4 cells were inoculated with ZIKV-WT, ZIKV-TL.PK, and ZIKV-p.2.5′ (MOI = 0.1) followed by harvest of supernatant for plaque assay analysis. *P < 0.0001, Two-way ANOVA. *N* = 3 per group/time point.

Supernatant from all samples was also analyzed by plaque-forming unit (PFU) assay to measure production of new infectious virus during infection. At 24 hpi, ZIKV-TL.PK and ZIKV-p.2.5′ produced over 10-fold and 1,000-fold, respectively, less infectious virus compared to ZIKV-WT at the same time point (Fig. 2C). ZIKV-TL.PK virus production caught up to ZIKV-WT by 72 hpi, while ZIKV-p.2.5′ consistently produced 10-fold less infectious virus than ZIKV-WT and ZIKV-TL.PK at later time points. These data suggest that ZIKV DB mutant virion release, as measured by supernatant ZIKV positive-strand genome copies, is unchanged, but ZIKV DB mutant infectious virion production is decreased or attenuated in mammalian cells.

To understand the phenotypic effects of the TL.PK and p.2.5′ mutants in invertebrate cells, we inoculated U4.4 mosquito cells with ZIKV-WT, ZIKV-TL.PK, and ZIKV-p.2.5′ following the same conditions stated above. Again, we found that ZIKV-TL.PK and ZIKV-p.2.5′ exhibited unchanged positive-strand genome copy replication compared to ZIKV-WT in the supernatant and inside cells at all time points (Fig. 2D and E). As in mammalian cells, we found that production of infectious ZIKV was similar in U4.4 cells for ZIKV-WT and ZIKV-TL.PK isolates, and the ZIKV-TL.PK mutant catches up in infectious virus production at 72 h (Fig. 2F). ZIKV-p.2.5′ exhibited significant reduction in infectious virus titer production, was undetectable through 48 h, but increased at 72 h while still exhibiting a 50-fold reduction at 72 h compared to ZIKV-WT (Fig. 2F). These data support our findings in mammalian cells suggesting that ZIKV DB mutants exhibit attenuation by a mechanism independent of ZIKV positive-strand genome production.

### ZIKV-TL.PK and ZIKV-p.2.5′ exhibit decreased caspase-3 activation

In our evaluation of viral titer in the ZIKV DB mutants, we noted that the plaque morphology of ZIKV-TL.PK and ZIKV-p.2.5′ was altered due to reduced cell monolayer clearance, resulting in less well-defined plaque edges compared to ZIKV-WT (Fig. 3A). These data align with findings from previous studies that showed flaviviruses with 3′ UTR structural deletions produced reduced cytopathic effect and plaque sizes compared to WT virus (14, 20, 24 - 30). We next quantified the cytopathic effect of ZIKV-TL.PK and ZIKV-p.2.5′ clones in A549 cells using an CyQUANT XTT cell viability assay. A549 cells were inoculated with ZIKV-WT, ZIKV-TL.PK, and ZIKV-p.2.5′ (MOI = 0.1) and harvested at 24, 48, and 72 hpi for analysis. ZIKV-WT-infected cells exhibited a significant decrease in cell viability throughout all time points compared to mock-infected control cells (*P < 0.001, Fig. 3B). Cells infected with ZIKV-TL.PK exhibited an initial 24% reduction in viability compared to mock-infected control cells at 24 and 48 hpi. However, ZIKV-TL.PK-infected cells exhibited no significant difference in cell viability at 72 hpi compared to control cells and significantly increased cell survival compared to WT ZIKV-infected cells (*P < 0.0001, Fig. 3B). Cells infected with ZIKV-p.2.5′ mutants exhibited no significant change in cell viability compared to mock-infected cells throughout the time course (Fig. 3B).

**Figure:**
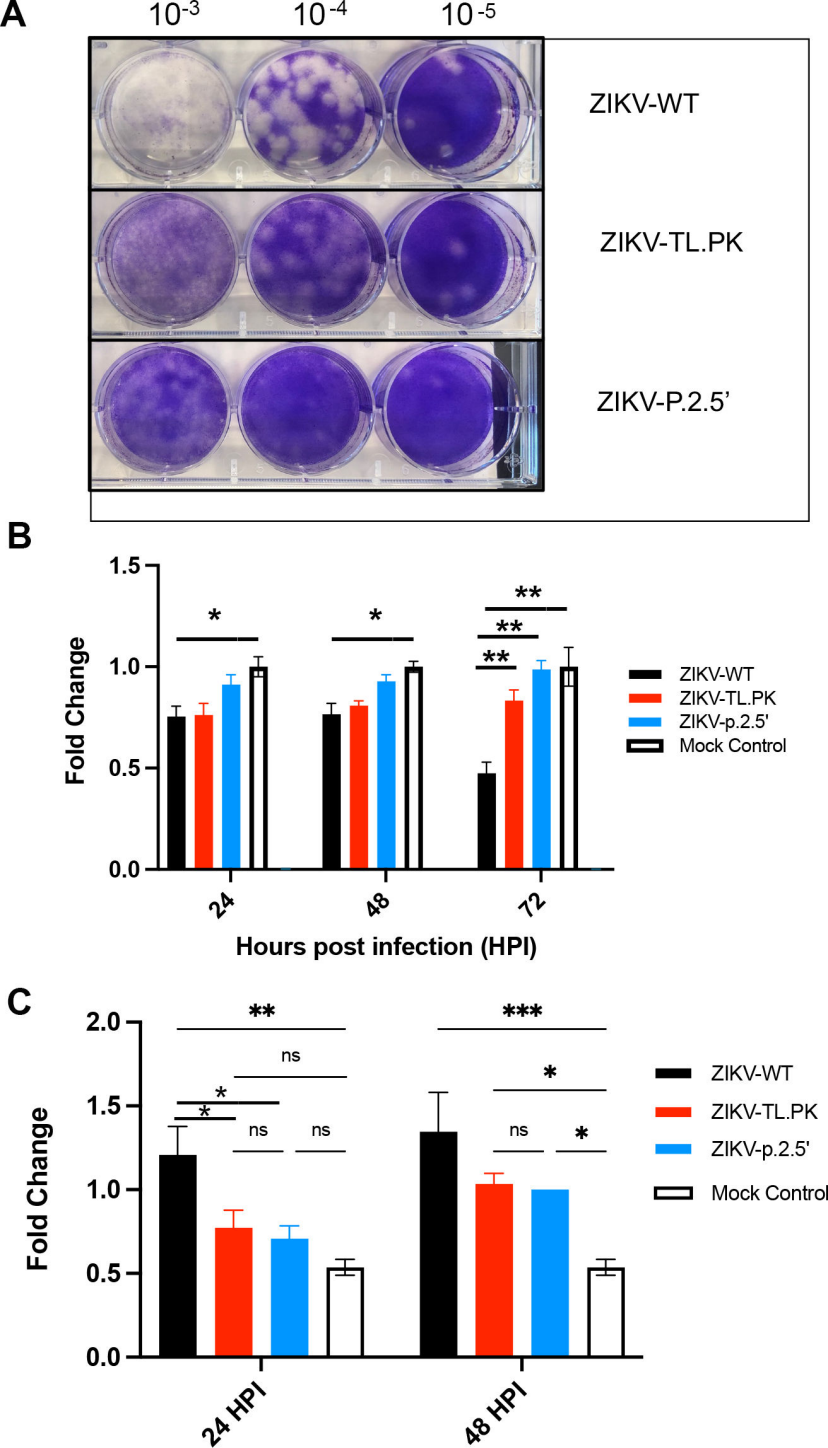
**FIG 3** ZIKV-TL.PK and ZIKV-p.2.5′ exhibit reduced pathogenesis due to reduced caspase-3 activation. (**A**) Image of representative plaque assays for ZIKV-WT, ZIKV-TL.PK, and ZIKV-p.2.5′ showing altered plaque phenotype in ZIKV DB mutants. A549 cells were inoculated with ZIKV-WT, ZIKV-TL.PK, and ZIKV-p.2.5’ (MOI = 0.1) and harvested at indicated time points for (B) XTT assay (*N* = 9 per group/time point) and (C) caspase-3 activity assay (*N* = 3 per group/time point). Two-way ANOVA, *P < 0.05; **P < 0.001; ***P < 0.0001; ns, not significant.

To define the mechanism by which ZIKV-TL.PK and ZIKV-p.2.5′ cause reduced cell injury, we inoculated A549 cells with ZIKV-WT, ZIKV-TL.PK, and ZIKV-p.2.5′ (MOI = 0.1) and evaluated caspase-3 and caspase-1 activation using caspase activity assays. Compared to ZIKV-WT inoculated cells, we found that ZIKV-TL.PK and ZIKV-p.2.5′ exhibited significantly less caspase-3 activation at 24 h post-infection (*P < 0.05) and no significant increase in caspase-3 activation compared to mock (Fig. 3C). At 48 h post-infection, ZIKV-TL.PK and ZIKV-p.2.5′ exhibited a significant increase in caspase-3 activity compared to mock-inoculated cells (*P* < 0.05) but still exhibited a non-significant 25% decrease in caspase-3 activity compared to WT ZIKV-infected cells. Next, we found no significant change in caspase-1 activation following infection in A549 cells with WT ZIKV or ZIKV DB-1 mutants (Fig. S3). These data show that ZIKV-TL.PK and ZIKV-p.2.5′ are attenuated due to decreased caspase-3-induced cell death following infection.

### ZIKV-TL.PK and ZIKV-p.2.5′ exhibit decreased sfRNA levels

sfRNA formation and function are essential to flavivirus cytopathic effect and pathogenesis (15, 26 - 30). In the ZIKV 3′ UTR, stem loops 1 and 2, also known as xrRNAs, are known to be crucial for the formation of sfRNAs (14). Previous studies using large deletions of 3′ UTR structures indicated that flavivirus DB structures may alter sfRNA levels in WNV, DENV, and ZIKV (19, 20, 24). We inoculated A549 cells with ZIKV-TL.PK and ZIKV-p.2.5′ isolates (MOI = 0.25) and harvested cellular RNA at 12, 24, and 48 hpi to assay sfRNA levels using Northern blot analysis with a probe specific for a conserved region of the ZIKV DB-1 structure. An MOI of 0.25 was selected as the highest possible MOI, given the virus titer of the ZIKV-p.2.5′ mutant virus isolates. In cells infected with ZIKV-WT, sfRNA bands start appearing at 12 hpi, with bands for sfRNAs 1, 2, and 3 at 24 and 48 hpi (Fig. 4A). However, the ZIKV-TL.PK mutant exhibited reduced levels of all sfRNAs at 24 and 48 hpi compared to WT. ZIKV-p.2.5′ exhibited no visible sfRNA levels at 12 or 24 hpi, and faint bands for sfRNAs 1 and 2 at 48 hpi (Fig. 4A and B). We also found no evidence of sfRNA3 levels in either ZIKV-TL.PK or ZIKV-p.2.5′. These data show that mutation of the DB-1, 3D RNA structure results in marked decrease of sfRNA levels during infection despite the fact that ZIKV positive-strand genome replication was not significantly altered during infection.

**Figure:**
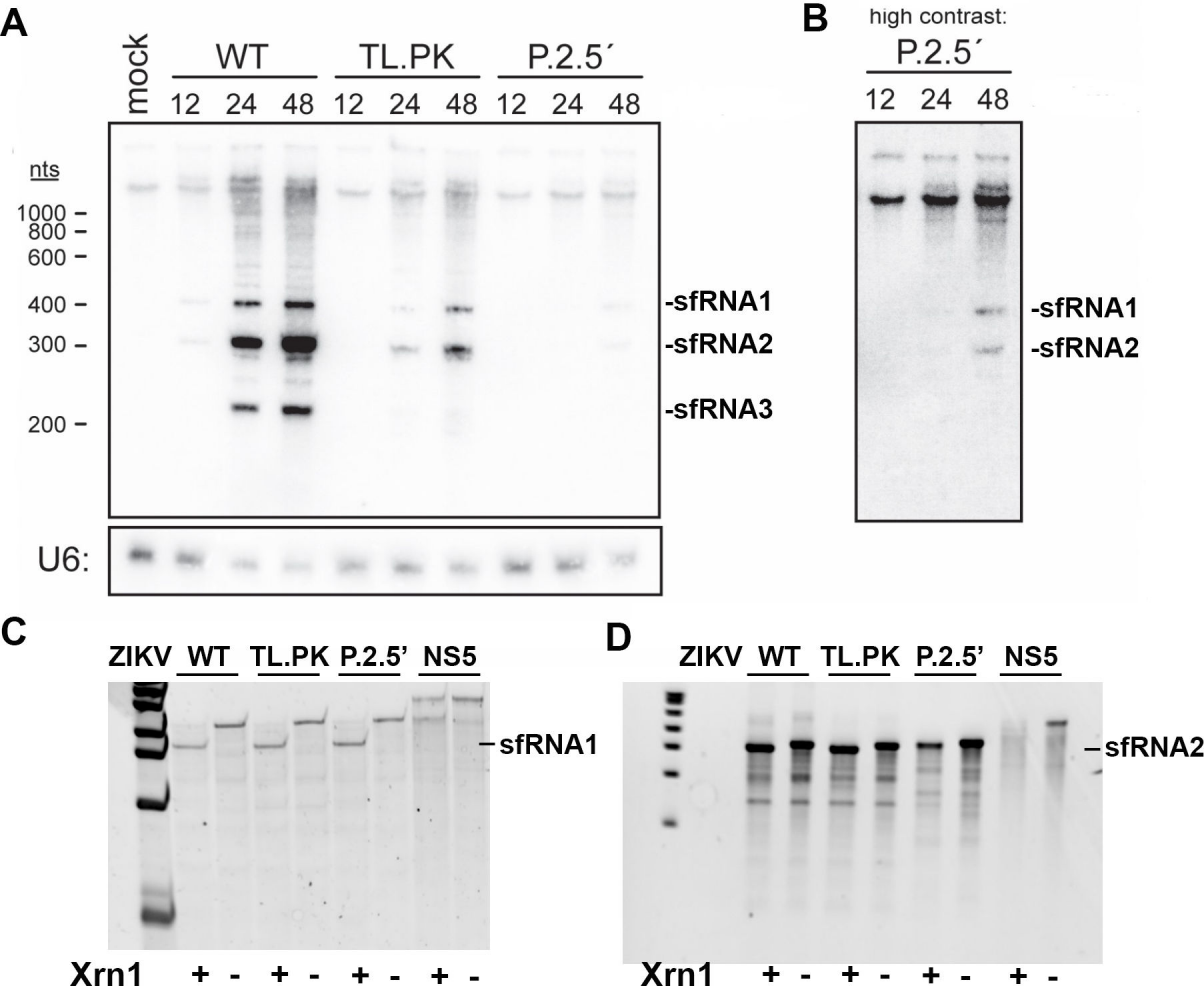
**FIG 4** ZIKV DB mutations result in altered sfRNA expression. A549 cells were inoculated with ZIKV-WT, ZIKV-TL.PK, and ZIKV-p.2.5′ (MOI = 0.1), and cells were harvested at indicated time points for (A) Northern blot analysis of sfRNA biogenesis at indicated hours post-infection. (**B**) Enhanced image of ZIKV-p.2.5′ mutant Northern blot image. Next, Xrn-1 digest assay of (C) full-length 3′ UTR and (D) xrRNA1 deleted 3′ UTR from ZIKV-WT, ZIKV-TL.PK, ZIKV-p.2.5′, and RNA from ZIKV NS5 gene as a control. Representative image of *N* = 2 per group.

Since sfRNA levels represent a balance between Xrn-1-dependent biogenesis and sfRNA degradation, we next investigated whether ZIKV-TL.PK and ZIKV-p.2.5′ cause a loss of Xrn-1 resistance during infection resulting in decreased biogenesis. We performed an *in vitro* Xrn-1 digest of 3′ UTR sequences containing either the WT, TL.PK, or p.2.5′ DB-1 3′ UTR sequences. We found no significant difference in production of sfRNA1 following Xrn-1 digest of 3′ UTR sequences from ZIKV-TL.PK and ZIKV-p.2.5′ compared to ZIKV-WT (Fig. 4C). Next, we produced a 3′ UTR sequence with xrRNA1 deleted and leaving downstream xrRNA2 followed by a 3′ UTR sequence from ZIKV-TL.PK and ZIKV-p.2.5′ compared to ZIKV-WT. We found no significant difference in production of sfRNA2 following Xrn-1 digest of xrRNA1 deleted 3′ UTR sequences from ZIKV-TL.PK and ZIKV-p.2.5′ compared to ZIKV-WT (Fig. 4D). These data show that biogenesis of xrRNA1 and 2-dependent sfRNA1 and 2 is unchanged in ZIKV-TL.PK and ZIKV-p.2.5′ mutants, but overall sfRNA levels are decreased during infection which implies the DB may play a role in preventing sfRNA decay following biogenesis.

In eukaryotes, 3′-to-5′ RNA degradation is mainly performed by the RNA exosome, with 3′ hydrolytic activity carried out by Rrp44 (31). Next, we investigated 3′-end exonuclease resistance of the ZIKV 3′ UTR and determined if the ZIKV DB-1 contributes to inhibition of 3′ end processing following biogenesis. We used *in vitro* digests of whole-length 3′ UTR WT, TL.PK, and p.2.5′ constructs with RNaseR. RNaseR is a commercially available *Escherichia coli* 3′ exonuclease homologous to the eukaryotic Rrp44 (31). We completed an *in vitro* RNaseR digest of 3′ UTR sequences containing either the WT, TL.PK, or p.2.5′ DB-1 3′ UTR sequences with and without the terminal 3′ stem loop which is known to inhibit 3′ end processing. We found that ZIKV wild-type and DB mutants exhibit no significant resistance to 3′ end processing in the absence of the terminal 3′ stem loop (Fig. S4). These data suggest that the terminal 3′ stem loop in the flavivirus 3′ UTR is the primary site 3′ exonuclease inhibition, and the 3′ UTR DBs exhibit no resistance to 3′-end exonuclease digest either alone or in cooperation with other 3′ UTR RNA structures.

### Mechanism of ZIKV-TL.PK and ZIKV-p.2.5′ attenuation

sfRNAs have a myriad of functions during flavivirus infection, including contributing to viral cytopathic effect. Since ZIKV-TL.PK and ZIKV-p.2.5′ clones exhibited decreased sfRNA levels during infection, we wanted to investigate which sfRNA functions were associated with the observed loss of cytopathic effect and caspase-3 activation in the ZIKV DB mutants. An important function of sfRNAs during infection is to antagonize the type I interferon responses to promote viral immune escape and viral replication (26 - 30 - 32 - 35). It has been shown in previous studies that flaviviruses lacking sfRNAs are more susceptible to T1IFN inhibition during infection (25, 36). Since ZIKV-TL.PK and ZIKV-p.2.5′ exhibited reduced sfRNA levels compared to ZIKV-WT, we next evaluated sensitivity of ZIKV growth to T1IFN treatment. We infected Vero cells with ZIKV-WT, ZIKV-TL.PK, and ZIKV-p.2.5′ (MOI = 0.1) followed by treatment with a dilution series of IFN-β and harvested cells at 48 hpi for viral positive-strand genome quantification by RT-qPCR. We found that positive-strand genome replication of ZIKV-TL.PK (IC50 = 99.6 IU/mL) was not significantly more sensitive to interferon compared to ZIKV-WT (IC50 = 96.1 IU/mL). However, we found that ZIKV-p.2.5′ (IC50 = 50.07 IU/mL) was significantly more sensitive to interferon treatment compared to ZIKV-WT (Fig. 5A; Fig. S5A).

**Figure:**
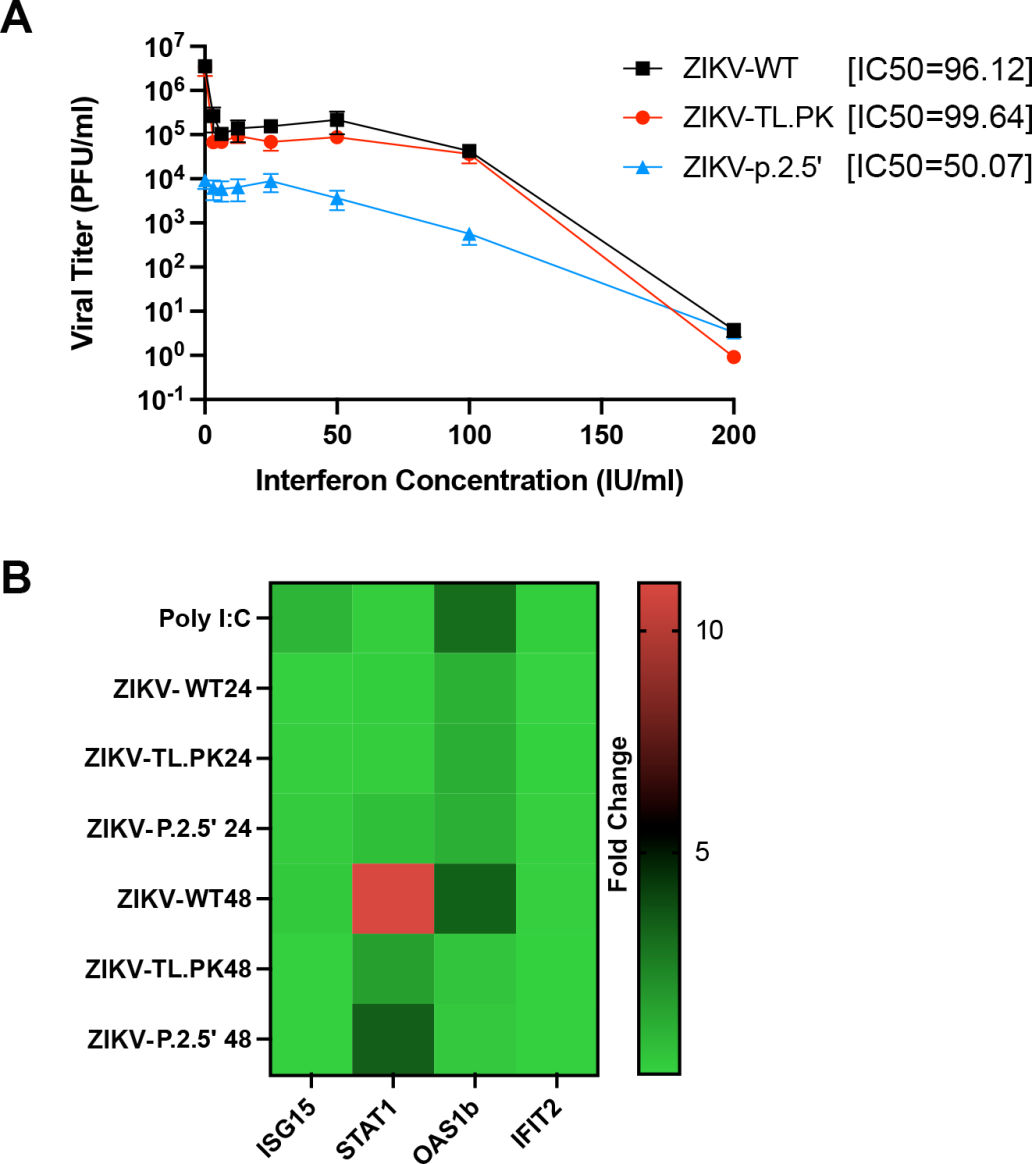
**FIG 5** ZIKV DB mutant viruses exhibit increased sensitivity to interferon independent of interferon stimulated gene expression. (**A**) Vero cells were inoculated with ZIKV-WT, ZIKV-TL.PK, and ZIKV-p.2.5′ (MOI = 0.1) followed by treatment with type I interferon (beta) at indicated concentrations and harvested at 48 h post-infection for plaque assay analysis. *N* = 6 per virus/interferon treatment group. Calculated type I interferon IC50 for ZIKV-WT (96.12), ZIKV-TL.PK (99.64), and ZIKV-p.2.5′ (50.07). (**B**) A549 cells were inoculated with ZIKV-WT, ZIKV-TL.PK, and ZIKV-p.2.5′ (MOI = 0.1) and harvested at 24 and 48 h post-infection for RT-PCR array for interferon-stimulated gene expression (*N* = 6 per group). Fold change (ddCT) calculated compared to mock-inoculated A549 cells.

The presence of sfRNAs can also significantly influence the induction of interferon stimulated genes (ISGs) during infection. sfRNA-deficient ZIKV has been shown to result in altered mRNA expression of several ISGs following infection (37). We next investigated ISG expression following infection with ZIKV-WT, ZIKV-TL.PK, or ZIKV-p.2.5′ mutants in mammalian cells to determine if ZIKV DB-1 mutants induced altered ISG expression. A549 cells were inoculated with ZIKV-WT, ZIKV-TL.PK, or ZIKV-p.2.5′ (MOI = 0.01) and harvested 24 and 48 hpi for total RNA. Total RNA was first evaluated for ISG expression at 24 hpi using a PCR array. We found no significant increases in ISG expression in ZIKV-WT, ZIKV-TL.PK or ZIKV-p.2.5′ compared to mock-infected cells at 24 hpi; however, several ISGs including ISG15, STAT, and IFIT exhibited potential trends in expression changes (Fig. S5B). Next, we assayed the treatment groups above at 24 and 48 hpi using RT-qPCR to evaluate mRNA expression levels for ISG15, STAT1, Oas1b, and IFIT2. At 48 hpi, we found that ZIKV-WT exhibited significantly increased gene expression of STAT1 and OAS1b compared to mock-infected controls; while ZIKV-TL.PK and ZIKV-p.2.5′ exhibited decreased gene expression of STAT1 and Oas1b (Fig. 5B). These data show that ZIKV DB mutants exhibit decreased activation of specific ISGs compared to ZIKV-WT at 48 h post-infection.

### ZIKV-TL.PK and ZIKV-p.2.5′ pathogenesis in a murine model

Studies have shown that mutations and RNA structure-altering mutations in the flavivirus 3′ UTR can reduce viral burden and pathogenesis in mouse models (14, 25, 30, 36, 38 - 40). To investigate the pathogenesis of ZIKV DB-1 mutant clones in a mouse model, we used the interferon-gamma receptor1/interferon-alpha receptor1 knockout (AG129) mouse model for ZIKV infection as previously described (41). Mice were inoculated with 10^4^ PFU via intraperitoneal injection with ZIKV-WT, ZIKV-TL.PK, ZIKV-p.2.5′, or PBS and followed post-infection for weight loss and development of neuroinvasive disease. Mice were sacrificed when moribund over a 16 days time course. We found that ZIKV-TL.PK and ZIKV-p.2.5′-infected mice experienced significantly decreased weight loss compared to ZIKV-WT-infected mice (Fig. 6A through E). Next, a survival curve was completed with the same treatment groups that were followed post-infection until mice were sacrificed after exhibiting >15% weight loss or development of advanced neurologic disease. A calculated survival analysis (*n* = 6 per virus group) showed that ZIKV-TL.PK (median survival = 14.5 days) and ZIKV-p.2.5′ (median survival = 15.5 days) infected mice exhibited a significantly longer survival time compared to ZIKV-WT (median survival = 9 days) infected mice (*P < 0.0001, Fig. 6E).

**Figure:**
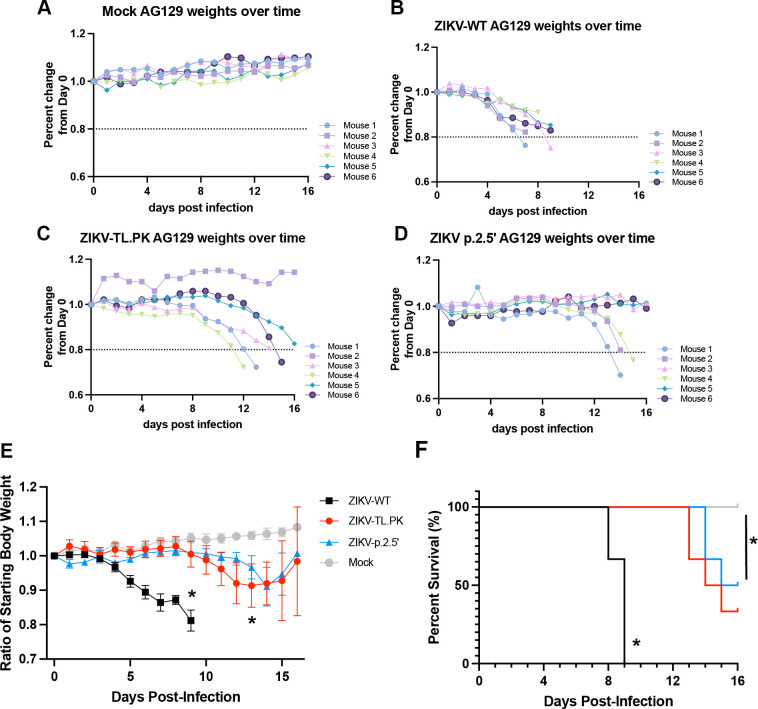
**FIG 6** ZIKV DB-1 mutant virus exhibits marked attenuation in AG129 mice. Mice were inoculated with ZIKV-WT, ZIKV-TL.PK, and ZIKV-p.2.5′ at 1,000 PFU by intraperitoneal injection. (**A–D**) Daily weights were recorded over 16 days for individual mice, and (**E**) mean data from each group analyzed for weight change over time post-infection (*P = 0.0023, mixed effects analysis). (**F**) Mice were inoculated with ZIKV-WT, ZIKV-TL.PK, and ZIKV-p.2.5′ at 1,000 PFU by intraperitoneal injection and euthanized when moribund (*P < 0.0001, log-rank test). *N* = 6 per group.

Next, AG129 mice were inoculated with ZIKV-WT, ZIKV-TL.PK, ZIKV-p.2.5′, or PBS as above and sacrificed 4, 6, or 8 days post-infection to analyze ZIKV positive-strand genome copies in the spleen and brain. We found no significant difference in positive-strand genome replication in the spleen when comparing ZIKV-WT to ZIKV-TL.PK or ZIKV-p.2.5 genome replication throughout the time course (Fig. 7A). However, we found a significant reduction (*P* = 0.03) in ZIKV-TL.PK and ZIKV-p.2.5 positive-strand genome replication in the brain compared to ZIKV-WT inoculated mice throughout the time course of infection (Fig. 7B). These data show that the ZIKV-TL.PK and ZIKV-p.2.5′ clones exhibit marked, end-organ specific attenuation in an immunocompromised murine model of ZIKV infection.

**Figure:**
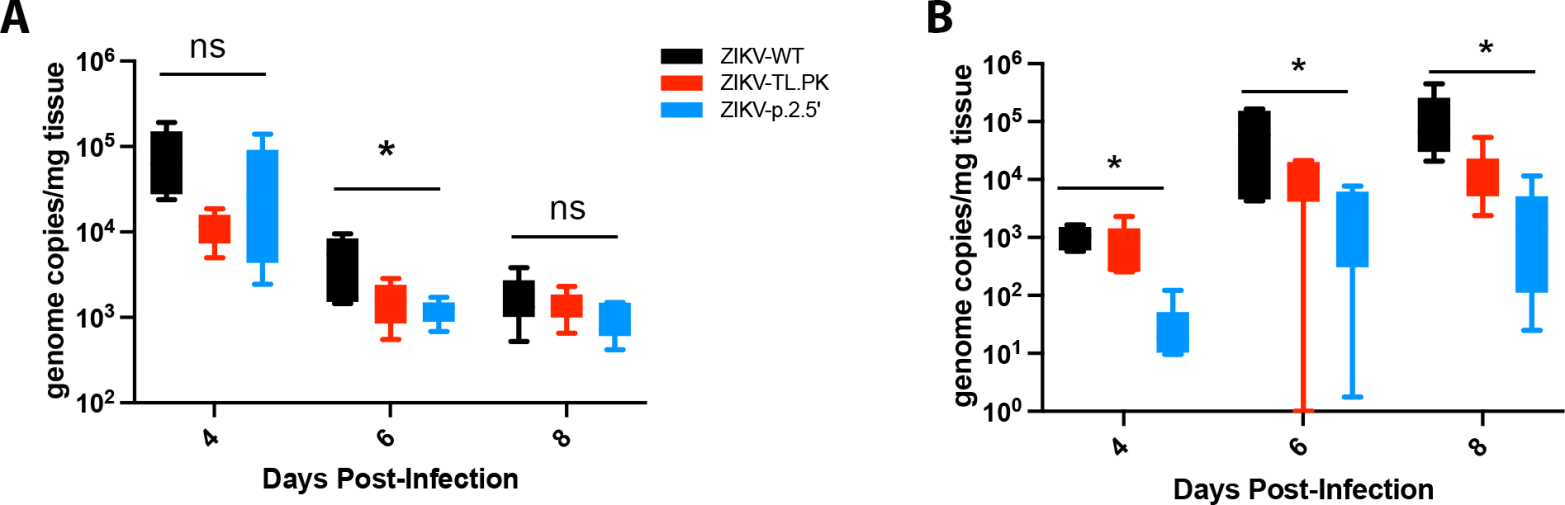
**FIG 7** ZIKV DB-1 mutant virus exhibits attenuated virus growth in the brain. Mice were inoculated with ZIKV-WT, ZIKV-TL.PK, and ZIKV-p.2.5′ at 1,000 PFU by intraperitoneal injection followed by tissue harvest at the indicated time point post-infection (*N* = 6 per group per time point). ZIKV genome copies were determined in the (A) spleen (*P = 0.0115) and (B) brain tissue at indicated days post-infection (*P < 0.048, one-way analysis of variance). ns, not significant.

## DISCUSSION

In this study, we sought to understand the contribution of the ZIKV 3′ UTR DB-1 to viral pathogenesis and define mechanisms of DB-1-dependent attenuation in flaviviruses. We utilized the recently solved DB-1, 3D structure from Donggang virus to guide targeted mutations that disrupt the secondary and tertiary structure of DB-1 (19), with the goal of eliminating the function of the DB-1 structure while maintaining the full-length sequence of the 3′ UTR. Based on previous studies indicating that flavivirus dumbbell structures regulate positive-strand genome replication (19, 42), we initially hypothesized that the ZIKV DB-1 structure was important for regulating viral positive-strand genome replication and that disruption if the ZIKV DB-1 structure would alter viral genome replication. The ZIKV positive-strand viral RNA is also the substrate for Xrn-1 degradation, and quantification of intracellular genome is important to understand associated changes in sfRNA expression. We found that ZIKV-TL.PK and ZIKV-p.2.5′ exhibited no significant change in viral positive-strand genome replication compared to ZIKV-WT virus. However, we did find that alteration of the flavivirus DB-1 structure resulted in reduced sfRNA levels during infection associated with decreased cell injury and decreased caspase-3-dependent cell injury in mammalian cells. Since sfRNA levels are a result of both biogenesis and decay, we evaluated the role of the ZIKV DB in sfRNA biogenesis using an *in vitro* Xrn-1 digest assay. Importantly, the decrease in ZIKV DB mutant sfRNA levels during infection occurs independent of xrRNA-dependent sfRNA biogenesis *in vitro*. Taken together, these data suggest a novel function for the flavivirus 3′ UTR DB-1 structure following sfRNA biogenesis in support of maintaining sfRNA levels during infection and potentially inhibiting sfRNA decay. However, the mechanism of ZIKV-DB-dependent support of sfRNA levels remains unclear.

Many flavivirus vaccine approaches include attenuating mutations and deletions that target either the 5′ UTR or the 3′ UTR (43 - 45). An important dengue vaccine candidate achieved attenuation in part due to a 30-base pair deletion in the 3′ UTR of the four serotypes of dengue virus, which includes 5′ portion of the dengue DB-1 region (44, 46). Despite the importance of this deletion in a structurally conserved region of the flavivirus 3′ UTR, the mechanism of attenuation is not clearly understood. Based on sequence analysis, the location of the deletion in the DB-1 region is likely to alter the structure of the DB without altering or changing genomic cyclization sequences critical for flavivirus genome replication and translation. Our data provide insight into mechanisms of attenuation associated with alterations in the flavivirus DB-1 structure. We found that DB-1 supports sfRNA levels in mammalian cells resulting in decreased caspase-3-induced cell death, increased sensitivity to type I interferon, and reduced pathogenicity as measured in both mammalian cells and in an immunocompromised murine model of ZIKV infection. Interestingly, the decrease in sfRNA levels by the ZIKV-p.2.5′ mutant was more pronounced than the ZIKV-TL.PK mutant, which was associated with increased attenuation as measured by the altered plaque phenotype, lack of cell injury as measured by XTT assay, increased interferon sensitivity, and decreased caspase-3 activation. This mechanism of attenuation in both ZIKV DB mutants is also associated with the phenotype of maintained ZIKV DB mutant particle production as measured by supernatant positive-strand RNA but decreased infectious titer of the ZIKV DB mutant in the supernatant of infected A549 cells. Taken together, we propose that our data provide important new insight into the mechanism by which flavivirus DB mutants result in attenuation and help guide rational design of future live-attenuated, flavivirus vaccine candidates through DB-1-specific targeted structural mutations.

sfRNAs are non-coding RNA made from the viral 3′ UTR after resisting host Xrn-1 degradation of the positive-strand viral RNA. Both xrRNA1 and xrRNA2 are the main structures responsible for 3′ UTR Xrn-1 resistance, and their 3D tertiary folding is critical for their ability to resist Xrn-1 degradation resulting in biogenesis of sfRNA1 and sfRNA2, respectively (14, 47, 48). Previous work to resolve the 3D RNA structure of the Donggang virus dumbbell found that while the DB forms a pseudoknot, it does not make the same loop around the 5′ end of the RNA to confer Xrn-1 resistance like xrRNA1 and xrRNA2 (19). Prior studies show that the ZIKV DB-1 does not efficiently resist Xrn-1 degradation on its own and that sfRNA3 may be generated by additional endonuclease or exonuclease processing (19). In this study, the ZIKV-TL.PK and ZIKV-p.2.5′ clones exhibited a significant decrease in levels of all sfRNA species compared to ZIKV-WT, and both clones exhibited loss of sfRNA3 levels. We also found that *in vitro* Xrn-1 digestion of ZIKV-TL.PK and ZIKV-p.2.5′ 3′ UTR sequences exhibited no changes in sfRNA1 and 2 biogenesis. We also found that the ZIKV DB does not exhibit resistance to 3′ exonuclease degradation from RNaseR, and the terminal 3′ stem loop of the 3′ UTR is the primary site of resistance. Together, our findings suggest that DB-1 plays a neutral role in Xrn-1-mediated sfRNA biogenesis such that structural changes that disrupt the 3D structure of DB-1 do not alter sfRNA biogenesis. Instead, disruption of the flavivirus 3′ UTR dumbbell structure likely alters interactions following biogenesis that may increase sfRNA decay or alter virus or host-protein interactions required to maintain sfRNA levels following biogenesis. Additional studies examining the interaction of flavivirus DB interactions within the generated sfRNA with host and viral factors may provide new insights into factors that contribute to sfRNA levels following biogenesis.

Our studies with the ZIKV-p.2.5′ clone also show for the first time that alteration of the 3D flavivirus DB-1 structure decreases sfRNA levels resulting in increased sensitivity to T1IFN treatment. In studies that deleted the flavivirus 3′ UTR DB, these viruses exhibited altered sfRNA patterns and exhibited increased sensitivity to T1IFN (20). WNV and YFV lacking sfRNAs replicated better in cells lacking factors in the T1IFN response than cells competent for all T1IIFN factors (20, 36). Interestingly, our data show that the ZIKV-TL.PK clone did not exhibit significantly altered IFN sensitivity compared to ZIKV-WT; however, the ZIKV-p.2.5′ exhibited significantly increased IFN sensitivity compared to wild-type virus. While both clones exhibit significantly less sfRNA levels compared to ZIKV-WT, the ZIKV-p.2.5′ exhibited a greater reduction in sfRNA levels than the ZIKV-TL.PK clone. It may be that even low levels of sfRNA during ZIKV-TL.PK infection were able to antagonize T1IFN responses, whereas ZIKV-p.2.5′ sfRNA levels are below a threshold needed to antagonize the interferon response. More studies comparing the sfRNA expression levels with interferon signaling are needed to define a potential dose-effect for sfRNA production to alter T1IFN signaling.

To evaluate the attenuation of the ZIKV-TL.PK and ZIKV-p.2.5′ in a murine model of ZIKV infection, we inoculated AG129 mice and determined survival and ZIKV positive-strand genome copies post-infection. These data show marked attenuation of the ZIKV-TL.PK and ZIKV-p.2.5′ as evident by significantly reduced weight loss and prolonged survival following infection. While ZIKV-TL.PK and ZIKV-p.2.5′ clones replicated in the spleen to similar levels as ZIKV-WT, viral genome copies of ZIKV-TL.PK and ZIKV-p.2.5′ clones were significantly decreased in the brain. These data imply that mutation of the DB-1 structure and subsequent decrease in sfRNA levels result in decreased replication in the brain as a mechanism for the decreased mortality seen in this murine model. Future studies will need to determine virus-specific neutralizing antibody responses and T-cell responses induced by ZIKV-TL.PK and ZIKV-p.2.5′. Further studies to determine attenuation and immunogenicity of DB mutants may provide a common attenuation approach for vaccine development against mosquito-borne flaviviruses by disrupting the DB-1 structure in the 3′ UTR.

## MATERIALS AND METHODS

### Cell lines and viruses

Mammalian Vero cells, mammalian A549 cells, and mosquito C6/36 cells were sourced from ATCC. Mosquito U4.4 cells were generously provided by Dr. Aaron Brault (CDC Center for Vector-Borne Diseases). Vero and C6/36 cells were cultured in 1× Minimal Essential Media (MEM; ThermoFisher, Denver, CO, USA), and A549 cells were cultured in Ham’s F-12 Nutrient Mix (ThermoFisher). Media for Vero, C6/36, and A549 cells were supplemented with 10% FBS, 1× MEM non-essential amino acids (ThermoFisher), 100 µM sodium pyruvate (ThermoFisher), 1 mM HEPES (ThermoFisher), and 1× penicillin/streptomycin (ThermoFisher, Denver, CO, USA). U4.4 cells were cultured in Mitsuhashi and Maramorosch Insect Media and supplemented with 10% FBS, 1× MEM non-essential amino acids, and 1× penicillin/streptomycin. Mammalian cell lines were cultured at 37°C with 5% CO_2_. Mosquito cell lines were cultured at 28°C with 5% CO_2_. Viruses used in this study are a wild-type ZIKV clone derived from the PRVABC59 ZIKV isolate, as well as two clone-derived ZIKV mutant strains (49).

### Plasmids and generation of TL.PK and p.2.5′ mutants

Previously described pACYC177 vector plasmids containing ZIKV PRVABC59 genome from either the 5′ UTR to nt 3498 (pJW231) or from nt 3109 to the end of the 3′ UTR (pJW232) were used to generate WT, TL.PK, and p.2.5′ ZIKV clones (49). TL.PK and p.2.5′ mutations were cloned into the pJW232 plasmid with gBlocks (IDT, Boulder, CO, USA) using Gibson assembly. Mutant gBlock inserts and pJW232 vector were linearized and amplified using PCR (Table 1). Gibson assembly was performed with the NEB Gibson Assembly Master Mix (New England Biolabs, Ipswich, MA, USA) with a vector-to-insert ratio of 1:5. Assembled plasmids were transformed into and isolated from NEB Stable Competent *E. Coli* cells (New England Biolabs) and amplified with rolling cycle amplification. Mutations were verified with Sanger sequencing (Eton Biosciences, San Diego, CA, USA).

**TABLE 1:** gBlock^*a*^ and primer sequences used for generating Zika virus mutants

Name	5′→ 3′ Sequence	Description
TL.PK gBlock	ggcgaccttccccacccttcaatctggggcctgaactggagatcaCGACAggatctccagaagagggactagtggttagaggagaccccccggaaaacgcaaaacagcatattgac	gBlock with TL.PK mutation sequence
p.2.5′ gBlock	ggcgaccttccccacccttcaatctggggcctgaaGACCTCTtcagctgtggatctccagaagagggactagtggttagaggagaccccccggaaaacgcaaaacagcatattgac	gBlock with p.2.5′ mutation sequence
p.1.5′ gBlock	ggcgaccttccccacccttcaatctggggcctgaaGACCTCTtcagctgtggatctccagaagagggactagtggttagaggagaccccccggaaaacgcaaaacagcatattgac	gBlock with p.1.5′ mutation sequence
MG-044	gcaggatgggaaaagaaggtggcgaccttccccacccttc	gBlock insert forward primer
MG-045	gagtctctggtctttcccagcgtcaatatgctgttttgcg	gBlock insert reverse primer
MG-046	cgcaaaacagcatattgacgctgggaaagaccagagactc	pJW231^*b*^ vector forward primer
MG-047	gaagggtggggaaggtcgccaccttcttttcccatcctgc	pJW231 vector reverse primer

^*a*^ gBlock =double-strand DNA fragment

^*b*^ pJW =plasmid JW

### Propagation and rescue of Zika virus clones

Wild type, TL.PK, and p.2.5′ pJW232 plasmids and wild-type pJW231 plasmid were digested and ligated together to create a DNA template of the complete ZIKV genome according to Sparks et al. (30). Wild type, TL.PK, and p.2.5′ genomic RNA were generated by *in vitro* transcription using the HiScribe T7 ARCA mRNA Kit (New England Biolabs). For ZIKV-WT, genomic RNA was electroporated into mosquito C6/36 cells using the following protocol: 8e^6^ cells were suspended in 400 µL of PBS with 40 µL of *in vitro* transcribed RNA. Cells were electroporated in a 0.2-cm cuvette with a square wave with 3 µF capacitance and 750 V for a 1 ms pulse. Cells were pulsed twice with 5 seconds between pulses. After electroporation, cells were rested at room temperature for 15 minutes.

For the DB-1 mutants, 40 µg of TL.PK and p.2.5′ genomic RNA was transfected into Vero cells using MessengerMAX Lipofectamine Reagent (Invitrogen). Virus was harvested when approximately 70% cell clearance was observed. Supernatant was spun down to clarify, and FBS and HEPES were added to supernatant to final concentrations of 20% and 10 mM, respectively. Virus was aliquoted and stored at −80°C. Extracellular viral RNA was quantified as described below in “Virus Quantification.” TL.PK and p.2.5′ Vero stocks were then blind passaged in C6/36 cells to generate higher titer virus. C6/36 cells were plated in T-182 flasks to sub-confluency. Volume of virus with a calculated value of 1e^8^ viral genomes was added to each flask. Virus was harvested when approximately 70% cell clearance was observed. Virus was harvested and stored as previously described. Viral titer was determined via plaque-forming unit assay as described in Virus Quantification.

### Virus quantification

The cell-free infection virus from the supernatant of infected cells was quantified using plaque-forming unit assay. Stock virus samples were serially diluted 10-fold 10^0^ to 10^−5^. An amount of 500 µL of each dilution was added to Vero cells plated in 6-well tissue culture plates to inoculated cells for 1 h at 37°C. After inoculation cells and viral inoculum were overlayed with a 1:1 mixture of 2.5% Avicel in DI-H_2_O and 2× MEM supplemented with 10% FBS, 10× sodium pyruvate, 10× non-essential amino acids, 10× penicillin-streptomycin, and 100 mM HEPES. Plaque assay plates were incubated for 6 days at 37°C. After incubation, cells were washed with 1 mL 1× PBS, then fixed with 4% paraformaldehyde for 15 minutes at room temperature. After PFA removal, plates were stained with 1% Crystal Violet in ethanol for 1 minute. Plates were washed three times with DI-H_2_O. Plaques were counted, and stock virus titer (PFU/mL) was calculated using the following equation:


(numberofplaques)×(dilutionfactor)×2=PFU/mL

Viral RNA was extracted from both the supernatant and cell lysate of infected cells with the E.Z.N.A. Viral RNA Kit (Omega Bio-Tek, Norcross, GA, USA) for L1-R studies and Luna RT for X2.L1 studies due to supply constraints for RT kits. Extracted RNA samples were then used to quantify the viral genome copies in each sample by RT-qPCR using the following protocol: (i) viral RNA was reverse transcribed using the iScript cDNA Synthesis Kit (Bio-Rad, Hercules, CA, USA); (ii) viral genome copies were quantified by qPCR using a combination of viral cDNA, Luna Universal Probe qPCR Master Mix (New England Biolabs), and the following primers in the envelope gene: Zika 1087: (5′-CCGCTGCCCAACACAAG-3′), Zika 1163c: (5′-CCACTAACGTTCTTTTGCAGACAT-3′), and FAM probe Zika 1108 FAM: (5′-AGCCTACCTTGACAAGCAGTCAGACACTCAA-3′).

### 3′ UTR sequencing

Viral genomic RNA for ZIKV-WT, TL.PK, and p.2.5′ was isolated from viral stocks using the Omega Bio-Tek E.Z.N.A. Viral RNA Kit. Using the linker sequence and reverse transcription primer described in Table 2, Viral cDNA was generated following the linker pre-adenylation, linker ligation, and reverse transcription protocols (19). 3′ UTR fragments were amplified using NEB Phusion Polymerase Kit with the following parameters: 98°C for 30 seconds; 30 cycles of: 98°C for 10 seconds, 58°C for 15 seconds, 72°C for 15 seconds; and 72°C for 10 minutes. Sanger sequencing was done by Eton BioSciences (San Diego, CA, USA).

**TABLE 2:** Linker and primer sequences for 3′ UTR sequencing

Name	5′ → 3′ Sequence	Description
ZIKV RNA-seq linker	(N_35_) TCTAGAGATCGGAAGAGCACACGTCTGAAddC	Linker sequence attached to 3′ end of ZIKV genome
ZIKV RNA-seq reverse primer	CTAGAGATCGGAAGAGCACAC	Primer for reverse transcription of the ZIKV genome; anneals to linker region
ZIKV Conserved 10455For	CAGGAGAAGCTGGGAAACC	Forward primer for ZIKV 3′ UTR amplification
ZIKV 10681Rev	AGTGGTTAGAGGAGACCCCC	Reverse primer for ZIKV 3′ UTR amplification

### *In vitro* viral growth kinetics

Mammalian A549 or mosquito U4.4 cells were seeded in 6-well plates at a density of 2e^5^ and 6e^4^ cells per well, respectively. Cells were infected with WT, TL.PK, or p.2.5′ virus at an MOI = 0.1. At 0, 24, 48, and 72 h post-infection, supernatant was harvested for quantification of viral genome copies by RT-qPCR and production of infectious virus by focus-forming unit assay. Infected cells were also harvested for quantification of cell-associated viral genome copies by RT-qPCR.

### XTT assay

A549 cells were plated in a 96-well clear bottom cell culture plate at a density of 10^4^ cells per well. Cells were infected with ZIKV-WT, TL.PK, and p.2.5′ virus at an MOI of 0.1 for 1 h at 37°C. Inoculum was removed, and 200 µL of complete Ham’s F-12 media was added to each well. XTT assay was performed with the XTT Cell Viability Kit from Cell Signaling Technology. At the harvest time point, control wells for 0% viability were treated with 100 µL 4% PFA for 15 minutes. Media in all wells was adjusted to 100 µL of complete Ham’s F-12 media. An amount of 50 µL of XTT detection solution was added to each well and incubated at 37°C for 4 h. After incubation, culture media in each well was treated with 100 µL 4% PFA for 15 minutes at room temperature to inactivated virus. Cell viability was quantified by measuring absorbance at 450 nm on a VersaMax Microplate Reader (Molecular Devices, San Jose, CA, USA).

### Caspase-1 and caspase-3 assays

10^6^ A549 cells were infected with ZIKV-WT, TL.PK, and p.2.5′ viruses at an MOI of 0.5 for 1 h at 37°C. After infection, inoculum was removed, cells were washed three times with 1 mL 1× PBS and 2 mL of complete Ham’s F-12 media. Samples were harvested at 24 and 48 h post-infection. Caspase activation was assayed with the caspase-3 and caspase-1 colorimetric assay kits from abcam (ab39401 and ab273268, respectively). At harvest, culture media was removed, and cells were washed one with 1× PBS. Cells were then scraped in 2 mL of 1 mL of 1× PBS and pelleted at 2,500 × *g* for 5 minutes at 4°C. Cell pellets were washed twice with 1 mL 1× PBS and pelleted again as previously stated. After the final wash, pellets were resuspended in 50 µL of cell lysis buffer and incubated on ice for 10 minutes. Cell debris was pelleted at 10,000 × *g* for 1 minute at 4°C. Lysate samples were flash frozen and stored at −80°C until time of assay. Lysate protein concentrations were quantified using the Pierce BCA Protein Assay Kit from ThermoFisher. Activated caspase-3 and caspase-1 were assayed following the manufacturer’s protocols from each caspase assay kit used.

### Northern blot

A549 cells were plated at a density of 2e^5^ cells per well in 6-well cell culture plates. Plates were infected with ZIKV-WT, TL.PK, and p.2.5′ viruses at an MOI of 0.5. Cells were infected with 500 µL of viral inoculum for 1 h at 37°C. After infection, inoculum was removed, cells were washed three times with 1 mL 1× PBS and 2 mL of complete Ham’s F-12 media. Samples were harvested at 0, 24, and 48 h post-infection. At harvest, media was aspirated, and cells were washed three times with 1× PBS. Three wells were combined for each strain at each time point to obtain sufficient levels of RNA. Total RNA was extracted from infected cells using the Omega Bio-Tek E.Z.N.A. Total RNA Kit I. RNA was extracted according to the manufacturer’s protocol. Northern blot for ZIKV sfRNA was performed according to Akiyama et al. (19).

### *In vitro* Xrn-1 digest of ZIKV 3′ UTR

Methods adapted from Slonchack et al. (29). ZIKV 3′ UTR templates were PCR amplified from the p2 plasmid using primers that include 100-nt of NS5 as an Xrn-1 leader sequence, plus a T7 RNA promoter sequence to the 5′ end of the template (Table 3). A control RNA was amplified from the NS5 gene upstream of the 3′ UTR-amplifying forward primer (Table 3). RNA was *in vitro* transcribed using the NEB HiScribe T7 Arca Kit, incubating the reaction for 1 h. Following *in vitro* transcription, RNA was purified using the Zymo RNA Clean and Concentrator Kit, eluting in 25 µL. An amount of 2 µg of RNA was folded in NEBuffer3 at 90°C for 3 minutes, followed by 20°C for 5 minutes. *In vitro* Xrn-1 digests were performed with 1 U Xrn-1 (New England Biolabs), 10 U RppH (New England Biolabs), and 1 U RNAseOUT (Invitrogen). Half of the RNA folding reaction was used in an Xrn-1 positive digest, while the other half was used for an Xrn-1 negative digest. Digests were incubated at 37°C for 2 h.

**TABLE 3:** Xrn-1 digest assay primers

Primer name	5′ → 3′ Sequence	Description
MG-052	taatacgactcactatagaggatcataggtgatgaagaaa	NS5-binding forward primer to amplify ZIKV 3′ UTR from pJW231 plasmid; adds T7 promoter sequence to 5′ end
MG-056	gtggggaaatccatgggtct	Reverse primer at 3′ end of ZIKV genome to amplify ZIKV 3′ UTR from pJW231 plasmid
Retcon Primer	taatacgactcactataggtggggaaatccatgggtct	NS5-binding forward primer for amplifying control sequence from NS5 from pJW231 plasmid; adds T7 promoter sequence to 5′ end
ZIKV Conserved 10321Rev	ataggtgatgaagaaaagtacatggac	NS5-bindng reverse primer for amplifying control sequence from NS5 from pJW231 plasmid

Digest products were analyzed by gel electrophoresis. A 5% TBE-Urea gel (Bio-Rad; Hercules, CA, USA) was pre-run at 200 V for 15 minutes in 1× TBE buffer. Xrn-1 digest samples were diluted 1:1 in 2× RNA loading dye (Bio-Rad), and RNA was denatured at 80°C for 90 seconds. Samples were run on the gel at 150 V for 45 minutes in 1× TBE. The gel was stained using 0.5 µg/mL ethidium bromide in 1× TBE for 30 minutes. Gels were imaged with a G:Box Gel Imager (Syngene; Frederick, MD, USA).

#### RNaseR digest of ZIKV 3′ UTR

A set of 3′ UTR DNA templates was prepared by PCR as described in the previous section, with another set of 3′ UTR DNA templates prepared by PCR to eliminate the 3′ SL of the 3′ UTR (Table 4). RNA was *in vitro* transcribed and folded according to the parameters described above. For one set of experiments, 2 µg of 3′ UTR constructs was folded in NEBuffer3 at 90°C for 3 minutes, followed by 20°C for 5 minutes. *In vitro* RNaseR digests were performed with 1 U RNaseR (Abcam, Waltham, MA, USA) and 1 U RNAseOUT (Invitrogen, Denver, CO, USA). Half of the RNA folding reaction was used in an RNaseR positive digest, while the other half was used for an RNaseR negative digest. Digests were incubated at 37°C for 2 h. For the other set of experiments, RNA was left unfolded, and 1 µg of template RNA was loaded directly into an RNaseR+ or RNaseR− digest reaction. Digests were incubated 37°C for 2 h.

**TABLE 4:** Primer sequences for *in vitro* RNaseR digest

Primer name	5′ → 3′ Sequence	Description
MG-052	taatacgactcactatagaggatcataggtgatgaagaaa	NS5-binding forward primer to amplify ZIKV 3′ UTR from pJW231 plasmid; adds T7 promoter sequence to 5′ end
MG-056	gtggggaaatccatgggtct	Reverse primer at 3′ end of ZIKV genome to amplify ZIKV 3′ UTR from pJW231 plasmid; used to generate full-length 3′ UTR
MG-045		Reverse primer upstream of 3′ UTR 3′ SL structure and downstream of DB-1; used to eliminate 3′ SL from 3′ UTR construct

Digest products were analyzed by gel electrophoresis. A 10% TBE-Urea gel (Bio-Rad) was pre-run at 200 V for 15 minutes in 1× TBE buffer. RNaseR digest samples were diluted 1:1 in 2× RNA loading dye (Bio-Rad), and RNA was denatured at 80°C for 90 seconds. Samples were run on the gel at 150 V for 45 minutes in 1× TBE buffer. The gel was stained using 0.5 µg/mL ethidium bromide in 1× TBE buffer for 30 minutes. Gels were imaged with a G:Box Gel Imager (Syngene).

### ISG RNA expression

A549 cells were infected at an MOI = 1 as described above. For poly I:C controls, 10^6^ were treated with 50 µg/mL of poly I:C in complete Ham’s F-12 media (described above). At 24 and 48 h post-infection or post-treatment, RNA was extracted from cells using the Omega Bio-tek E.Z.N.A. Total RNA Extraction Kit following manufacturer’s protocol. Whole cell RNA was reverse transcribed using the Bio-Rad gDNA Clear cDNA Synthesis Kit with 100 ng of RNA template and the addition of 1 µL of (RT control from plate) to each reaction. qPCR was done with Bio-Rad PrimePCR Interferon ⍺/β Signaling Pathway plates according to manufacturer’s protocol. Subsequent qPCR was completed for STAT1, ISG15, GAPDH, OAS1b, and IFIT2 using primers: STAT1 forward (AATATGTGGATGTCTCAGTGGT), STAT1 Rev (GGAAAACTGTCATCATAAAGCT), ISG15 For (CAG CGA ACT CAT CTT TGC CAG T), ISG15 Rev (CAT CTT CAC CGT CAG GTC CCA), GAPDH For (GAA GGC TGG GGC TCA TTT GC), GAPDH Rev (TAA GCA GTT GGT GCA GG), OAS1 For (GTGTATAATCCAGGACAGAACC), OAS1 Rev (GGCTGTGGAGAATGTTATCTAT), IFIT2 For (ACAAGCAGATGAAGACTCTGAG), and IFIT2 Rev (CACATCTCTATTCTTCATTCCC).

### Interferon dose-response curve

10^4^ Vero cells were infected with ZIKV-WT, -TL.PK, -p.2.5′, or mock infected at an MOI = 1 for 1 h at 37°C. After infection, cells were treated with IFN-β with a 1:2 dilution series from 100 to 3.125 IU/mL, including cells that went untreated. After 48 h, supernatant was harvested for viral RNA extraction. Viral RNA was extracted using the Omega Bio-tek E.Z.N.A. Viral RNA Extraction Kit. Viral genome copies in the supernatant were quantified using RT-qPCR as described above. Interferon-β treatments were done in triplicate for each virus condition.

### Animal studies

All animal and infectious disease studies were reviewed and approved by the University of Colorado Institutional Animal Care and Use Committee and Institutional Biosafety Committee. B6.CG-*Ifngr1^tm1Agt^Ifnar1^tm1.2Ees^*/J mice purchased from Jackson Laboratories (JAX stock #029098; Bar Harbor, Maine, USA) were maintained and bred in specific pathogen-free facilities at the University of Colorado Anschutz Medical Campus animal facility. Animals to be infected were housed in an animal BSL-3 (ABSL-3) facility. After infection, mice were observed daily for weight loss and signs of disease until the experiment endpoint.

### Murine experiments

Six- to eight-week-old mice were anesthetized with isoflurane and infected via intraperitoneal injection with 10^4^ PFU of virus. For mice assayed for survival curve, infected individuals were weighed daily until they reached 80% of their original weight at the time of infection. Mice were also monitored for other symptoms of illness, including hunched body, slow movement, and matted fur. Once mice showed significant illness and reached the weight cutoff, they were euthanized with isoflurane.

Animals infected for measuring viral load were euthanized with isoflurane 4, 6, or 8 days post-infection. Mice were perfused with 25–30 mL of 1× PBS, followed by dissection and harvest of spleen and brain. Organs were preserved in RNAlater solution until RNA extraction. RNA was extracted from tissue following the techniques used in reference (30). An amount of 1 µg of total organ RNA was reverse transcribed using the Bio-Rad iScript Reverse Transcription Kit according to manufacturer’s protocol. qPCR for Zika genome copies was performed as described above. Viral load was normalized by dividing the qPCR starting quantity value by the mass of harvested organ for each sample.

### Statistical analysis

Data were analyzed by two-way analysis of variance, assuming normal distribution. AG129 survival curve was analyzed using Kaplan-Meier graph. All statistical analysis done using GraphPad Prism.

## SUPPLEMENTAL MATERIAL

Figure S1 mbio.01108-23-s0001.tif Mfold RNA structure prediction of Zika virus DB mutants.

Figure S2 mbio.01108-23-s0002.tif Zika virus DB mutant sequencing results.

Figure S3 mbio.01108-23-s0003.tif Caspase-1 activity assay results.

Figure S4 mbio.01108-23-s0004.tif RNaseR *in vitro* degradation of Zika virus 3'UTR DB mutants.

Figure S5 mbio.01108-23-s0005.tif Interferon sensitivity and ISG expression analysis.

ASM does not own the copyrights to Supplemental Material that may be linked to, or accessed through, an article. The authors have granted ASM a non-exclusive, world-wide license to publish the Supplemental Material files. Please contact the corresponding author directly for reuse.
