# Supplementary figures and images for: Zika virus dumbbell-1 structure is critical for sfRNA presence and cytopathic effect during infection
Source: mBio. 2023 Jul 7;14(4):e01108-23. doi: 10.1128/mbio.01108-23 (PMC10470596; doi:10.1128/mbio.01108-23)

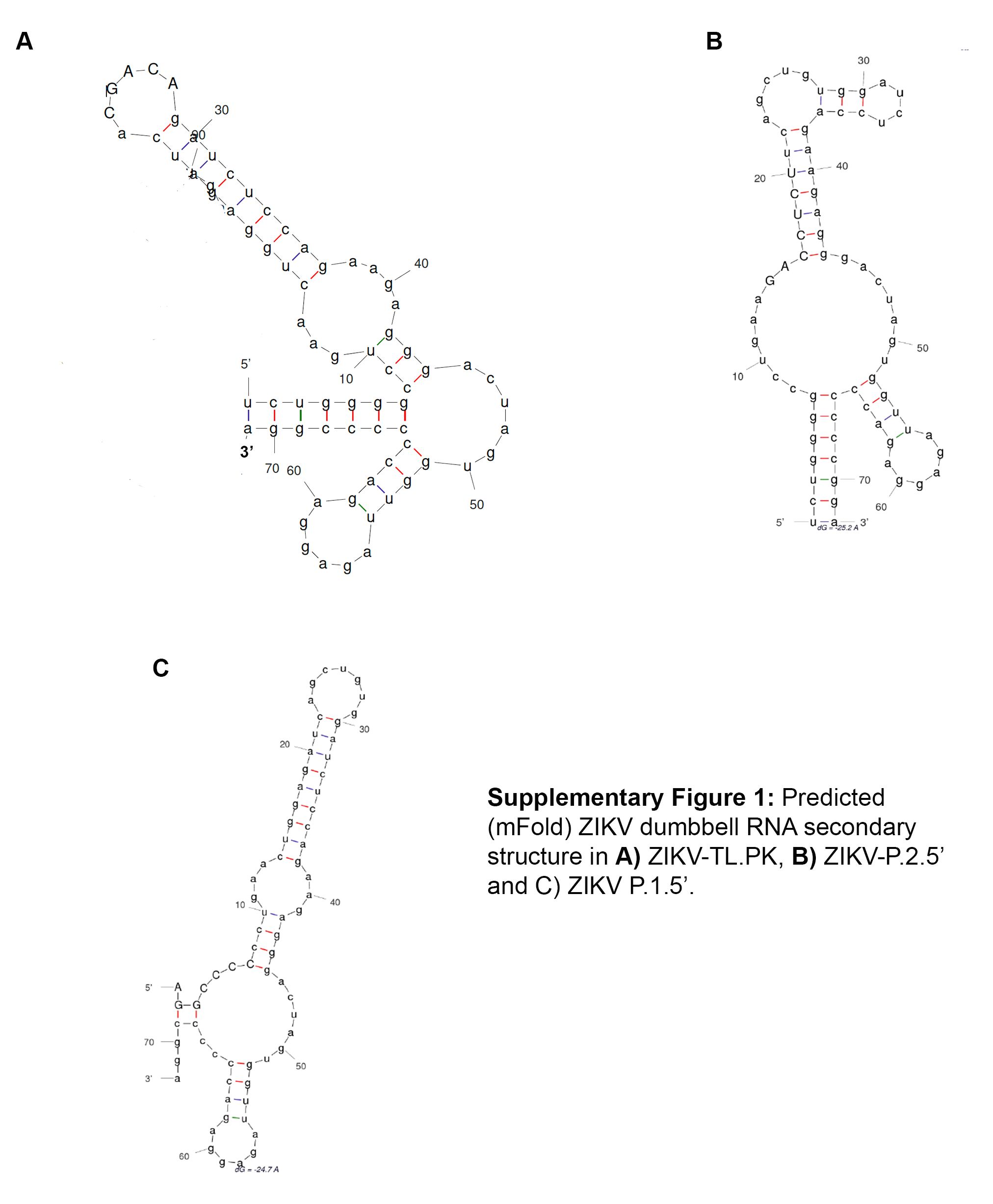

Supplement: Figure S1 — Mfold RNA structure prediction of Zika virus DB mutants. [file mbio.01108-23-s0001.tif]

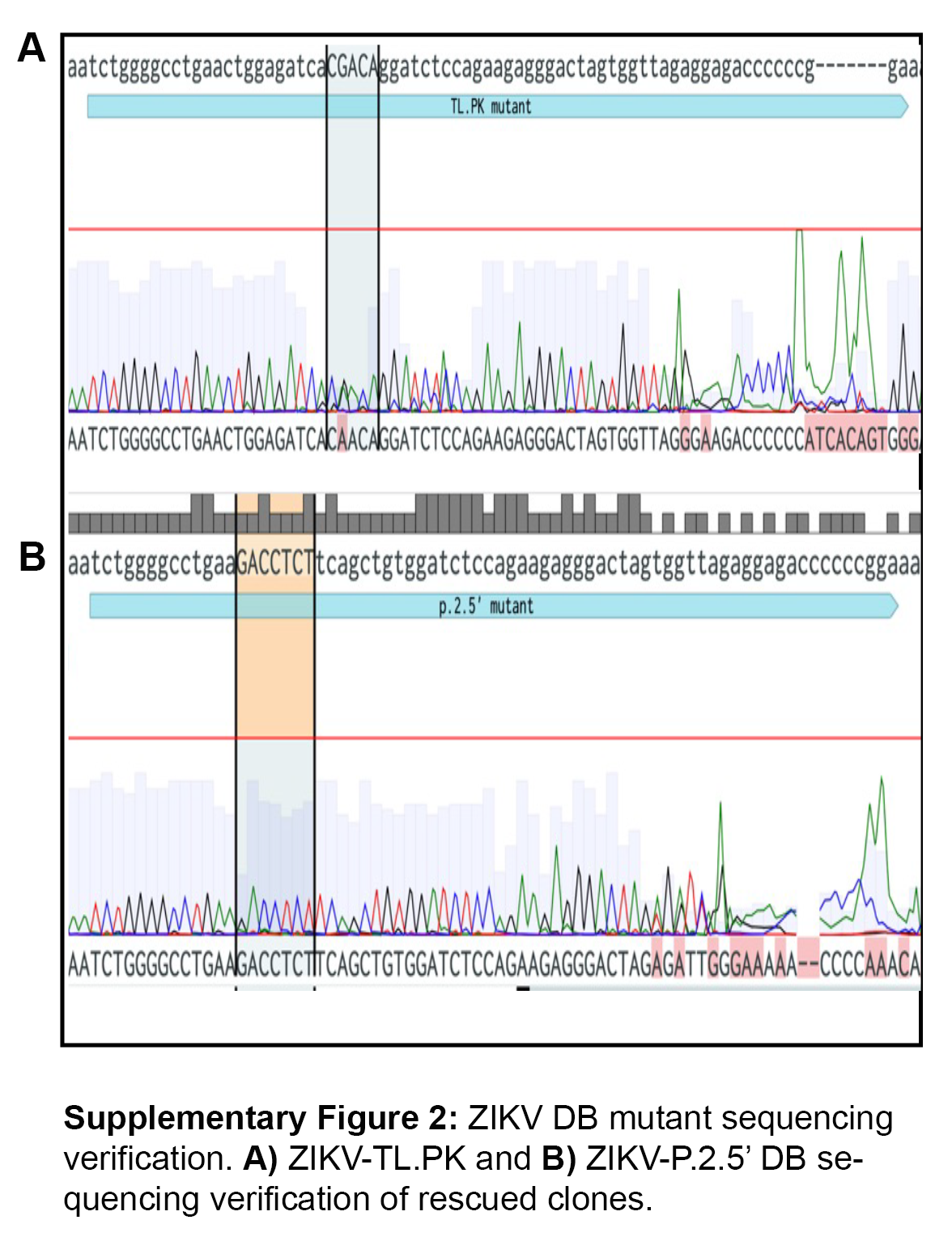

Supplement: Figure S2 — Zika virus DB mutant sequencing results. [file mbio.01108-23-s0002.tif]

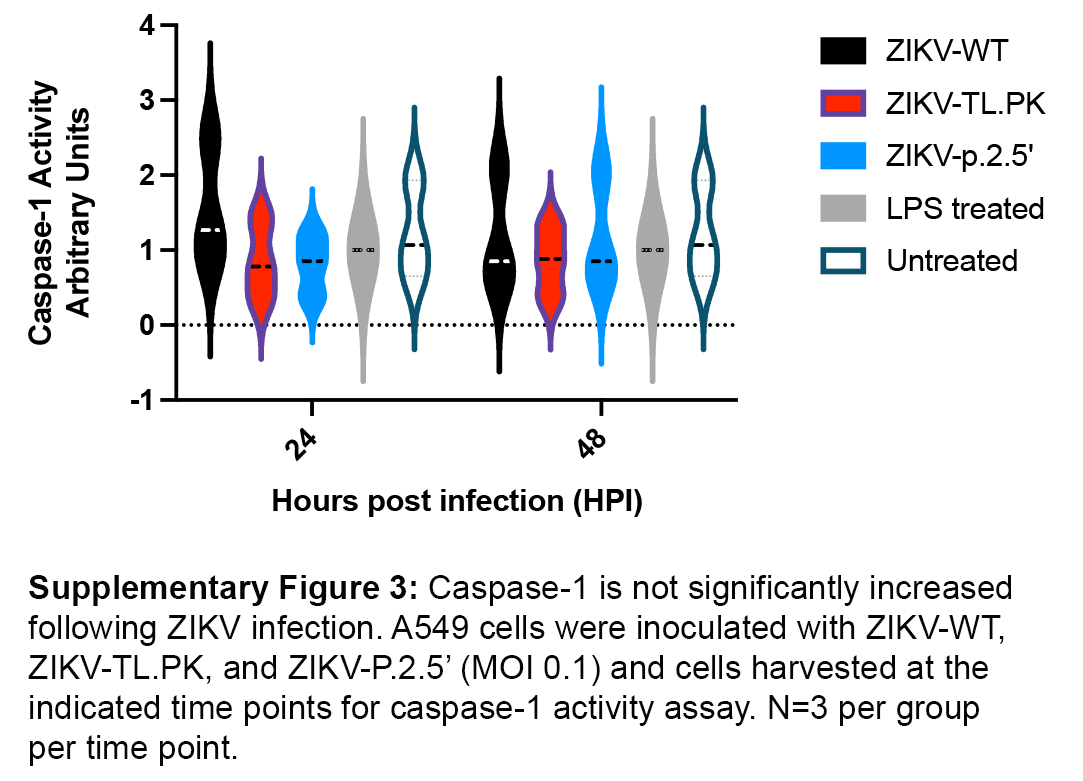

Supplement: Figure S3 — Caspase-1 activity assay results. [file mbio.01108-23-s0003.tif]

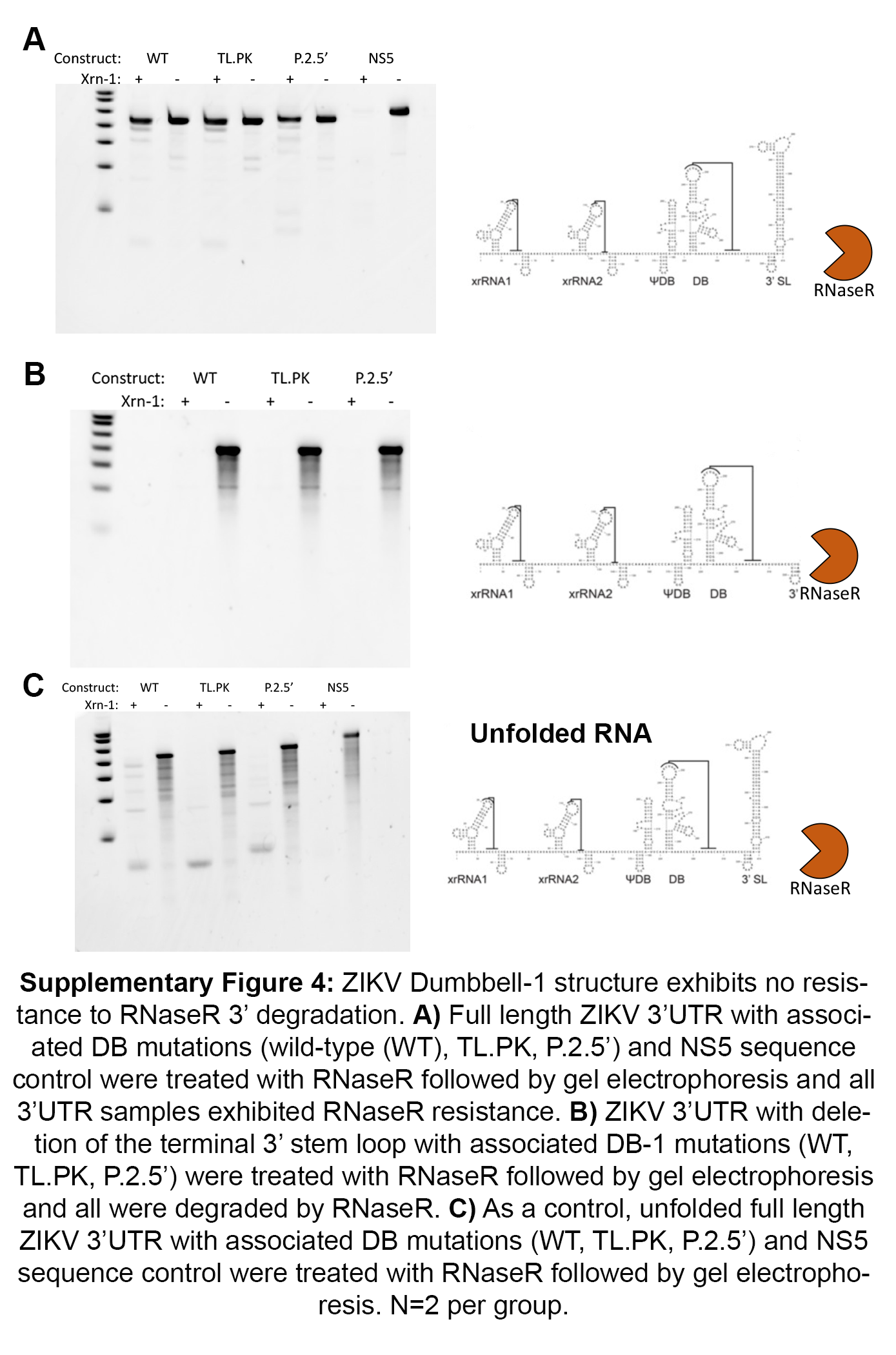

Supplement: Figure S4 — RNaseR in vitro degradation of Zika virus 3'UTR DB mutants. [file mbio.01108-23-s0004.tif]

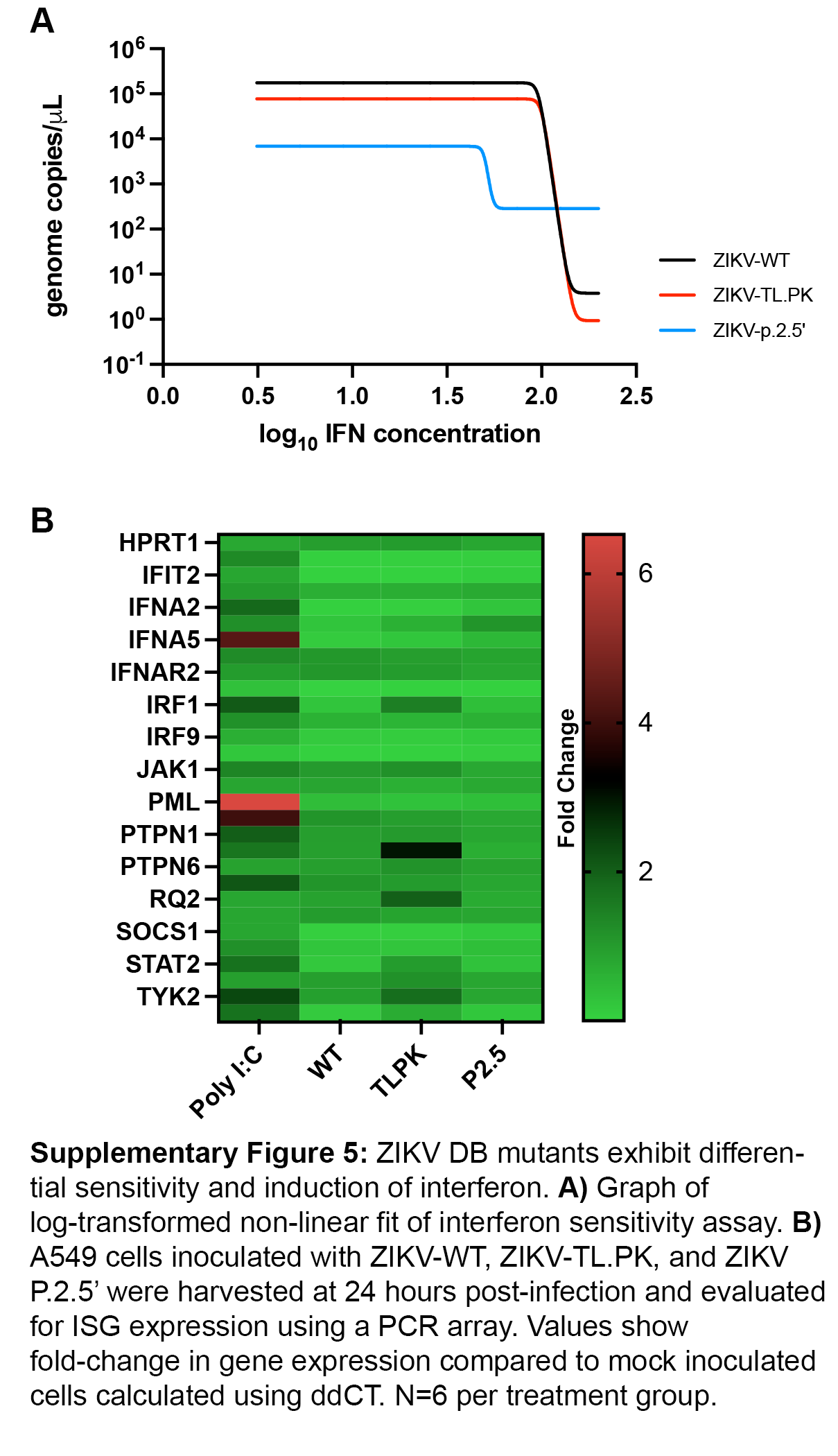

Supplement: Figure S5 — Interferon sensitivity and ISG expression analysis. [file mbio.01108-23-s0005.tif]
